# Predictors of Early Onset Multiple Organ Dysfunction in Major Burn Patients with Ventilator Support: Experience from A Mass Casualty Explosion

**DOI:** 10.1038/s41598-018-29158-3

**Published:** 2018-07-19

**Authors:** Jia-Yih Feng, Jung-Yien Chien, Kuo-Chin Kao, Cheng-Liang Tsai, Fang Ming Hung, Fan-Min Lin, Han-Chung Hu, Kun-Lun Huang, Chong-Jen Yu, Kuang-Yao Yang

**Affiliations:** 1 Department of Chest Medicine, Taipei Veterans General Hospital, Taipei, Taiwan; 2 School of Medicine, National Yang-Ming University, Taipei, Taiwan; 3 Institute of Clinical Medicine, School of Medicine, National Yang-Ming University, Taipei, Taiwan; 4 Department of Internal Medicine, National Taiwan University Hospital, National Taiwan University College of Medicine, Taipei, Taiwan; 5 Department of Thoracic Medicine, Chang Gung Memorial Hospital, Taoyuan, Taiwan; 6 Department of Respiratory Therapy, Chang Gung University, Taoyuan, Taiwan; 7 Division of Pulmonary and Critical Care, Department of Internal Medicine, Tri-Service General Hospital, National Defense Medical Center, Taipei, Taiwan; 8 Department of Surgical Intensive Care Unit, Far Eastern Memorial Hospital, New Taipei, Taiwan; 9 Division of Pulmonary Medicine, Department of Internal Medicine, Kaohsiung Armed Forces General Hospital, Kaohsiung, Taiwan; 10 Graduate Institute of Aerospace and Undersea Medicine, National Defense Medical Center, Taipei, Taiwan; 11 Institute of Emergency and Critical Care Medicine, School of Medicine, National Yang-Ming University, Taipei, Taiwan

## Abstract

Organ dysfunction is common in patients with major burns and associated with poor outcomes. The risk factors for early onset multiple organ dysfunction syndrome (MODS) in major burn patients with invasive ventilator support has rarely been evaluated before. In this study, major burn patients with invasive ventilator support from 499 victims suffered in a mass casualty color dust explosion were retrospectively enrolled. The development of early MODS that occurred within 5 days after burn injury was determined and the risk factors associated with early MODS were analyzed. A total of 88 patients from five medical centers were included. Their mean total body surface area (TBSA) was 60.9 ± 15.8%, and 45 (51.1%) patients had early MODS. Hematologic failure was the most common organ failure (68.6%), followed by respiratory failure (48.9%). Independent clinical factors associated with early MODS included TBSA ≥55% (OR: 3.83; 95% CI: 1.29–11.37) and serum albumin level <2.1 g/dL upon admission (OR: 3.43; 95% CI: 1.01–11.57). Patients with early MODS had prolonged ventilator dependence and longer ICU admission than those without early MODS. Our results showed that early MODS in major burn patients with invasive ventilator support is very common and can be predicted early on admission.

## Introduction

Major burn is one of the most severe forms of trauma and usually associated with high morbidity and mortality. Changes in metabolic status, such as hypermetabolism and catabolism, in patients with major burns can adversely affect organ function^1^. Several clinical factors have been proposed to predict the prognosis of burn patients, including age, total body surface area (TBSA) burned, and the presence of inhalation injury^2–5^. Meanwhile, the occurrence of organ dysfunction has also been proposed to be associated with treatment outcomes in major burn patients^6^.

Multiple organ dysfunction syndrome (MODS) is defined as the presence of dysfunction in two or more organs or organ systems induced by a variety of acute injuries. Although sepsis and septic shock are usually reported as the major causes of MODS^7,8^, this condition is also very common in victims with major burns and severe trauma^6,9^. Recent studies have demonstrated that the incidence of MODS in patients with trauma or burns ranges from 30% to 40%^10–13^. The hypermetabolic and catabolic responses after a major burn injury can impair the immune function and increase the risk for nosocomial infections and sepsis, which may in turn cause MODS^14,15^. Burn injuries can also induce microvascular hyperpermeability that leads to hemodynamic instability and organ damage^16^. The severity of MODS is one of the major determinants of the treatment outcomes of patients with major burns^17^. MODS is also a major cause of death if the patient survives within the first 24 hours^18^. Meanwhile, early organ dysfunction has a better correlation with mortality than late organ dysfunction in critically ill burn ill patients^6^.

Around 15% of patients with burns need invasive ventilator support, and their treatment outcomes could be even worse^19^. Considering the critical role of MODS in the treatment outcomes of patients with major burns, understanding the predictors of early MODS in patients with major burns would be helpful to clinicians in identifying high-risk patients who require the most intensive and aggressive management, particularly those with ventilator support. It is worth noting that most of the previous studies have included patients from different burn events. However, a mass casualty explosion occurred at a waterpark in Taiwan in 2015, and nearly 500 victims were reported^20^. In this retrospective observational study, we enrolled patients with major burns on invasive ventilator support from this event. The main purpose of this study was to investigate the predisposing factors for early MODS in this patient population. The impact of early MODS on the treatment outcomes, particularly ventilator weaning and mortality, were also assessed.

## Results

### Patient characteristics and burn severities

**Figure 1:**
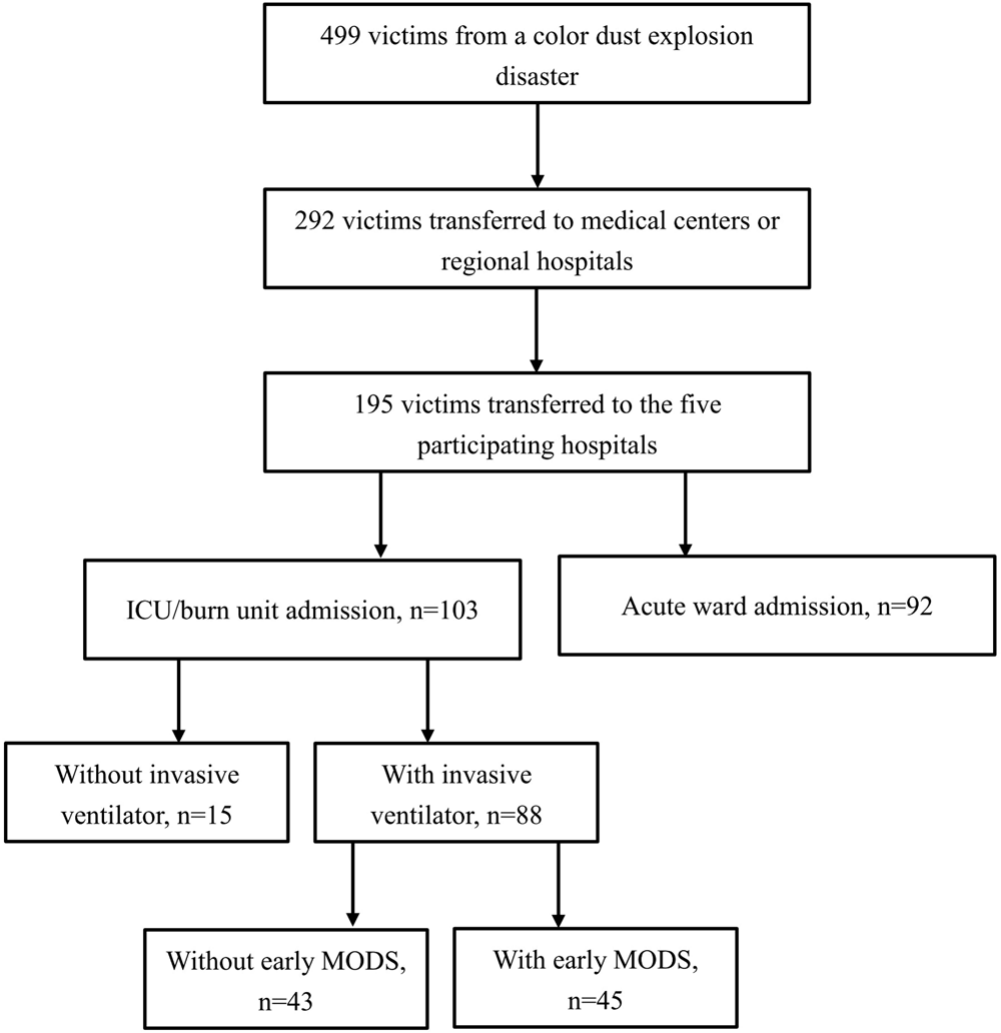
Study profile demonstrating the number of cases enrolled for analysis from a single explosion disaster.

During the study period, a total of 88 patients with major burns on invasive ventilator support were included in the analysis. The study profile showing the number of enrolment cases is shown in Fig. 1. The demographic characteristics and the burn severities of the patients are shown in Table 1. The sex distribution was equal, and the mean age was 21.3 ± 4.1 years. Their mean TBSA was 60.9 ± 15.8%, and 72.7% (64/88) of the patients had facial burns. Bronchoscopy was performed in 67 patients, of whom 17 (23.9%) had evidence of ≥grade 2 smoke inhalation injury. Meanwhile, we found a weak association between TBSA and serum albumin levels with a correlation coefficient of −0.366 (Supplementary Figure 1).

**Table 1:** Clinical characteristics and disease severities of patients with major burns on invasive ventilator support^a^.

	Overall, n = 88	Early MODS		P value
		No, n = 43	Yes, n = 45	
Age	21.3 (4.1)	21.4 (4.8)	21.2 (3.5)	0.825
Male gender	42 (44.7%)	18 (41.9%)	24 (53.3%)	0.281
Mean 2~3TBSA%	60.9 (15.8)	54.2 (15.2)	67.2 (13.7)	<0.001
Facial burn	64 (72.7%)	27 (62.8%)	37 (82.2%)	0.041
≥2^nd^ Smoke inhalation (n = 67)^b^	17 (23.9%)	9 (26.5%)	8 (21.6%)	0.632
Serum albumin (g/dL)	1.93 (0.69)	2.19 (0.82)	1.71 (0.44)	0.002
Organ failure				
Respiratory	43(48.9%)	12 (27.9%)	31 (68.9%)	<0.001
Cardiovascular	31 (35.2%)	7 (16.3%)	24 (53.3%)	<0.001
Hepatic	13 (14.8%)	2 (4.7%)	11 (24.5%)	0.009
Hematologic	59 (67.0%)	17 (39.5%)	42 (93.3%)	<0.001
Renal	8 (9.1%)	1 (2.3%)	7 (15.6%)	0.059
Metabolic	28 (31.8%)	6 (14.0%)	22 (48.9%)	<0.001
Coagulation	12 (13.6%)	3 (7.0%)	9 (20.0%)	0.075
No. of organ failure	2.25 (1.44)	1.12 (1.10)	3.33 (1.09)	<0.001
SOFA score				
Day 1	0.78 (1.09)	0.47 (0.77)	1.09 (1.26)	0.006
Day 3	2.91 (2.18)	1.93 (1.45)	3.84 (2.35)	<0.001
Day 7	4.95 (3.11)	3.28 (2.57)	6.56 (2.74)	<0.001
Day 14	4.15 (3.08)	2.81 (2.41)	5.42 (3.12)	<0.001
Day 21	2.84 (2.70)	1.98 (2.30)	3.67 (2.81)	0.003
Sputum culture				
GNB	34 (38.6%)	12 (27.9%)	22 (48.9%)	0.043
GPC	3 (3.4%)	2 (4.7%)	1(2.2%)	0.612
Fungus	5 (5.7%)	4 (9.3%)	1 (2.2%)	0.198
Bacteremia	21 (23.9%)	9 (20.9%)	12 (26.7%)	0.528
ECMO	5 (5.7%)	0	5 (11.1%)	0.056
Dialysis				
CVVH	6 (6.8%)	1 (2.3%)	5 (11.1%)	0.246
HD	1 (1.1%)	0	1 (2.2%)	1.000

^a^The data are presented as n (%) unless otherwise stated.

^b^Fiberbronchoscopy is optionally performed if clinically indicated.

TBSA, total body surface area; SOFA, Sequential Organ Failure Assessment; GNB, Gram-negative bacilli; GPC, Gram-positive cocci; ECMO, extracorporeal membrane oxygenation; CVVH, continuous venousvenous hemofiltration; HD, hemodialysis.

### Organ failure and early MODS

**Figure 2:**
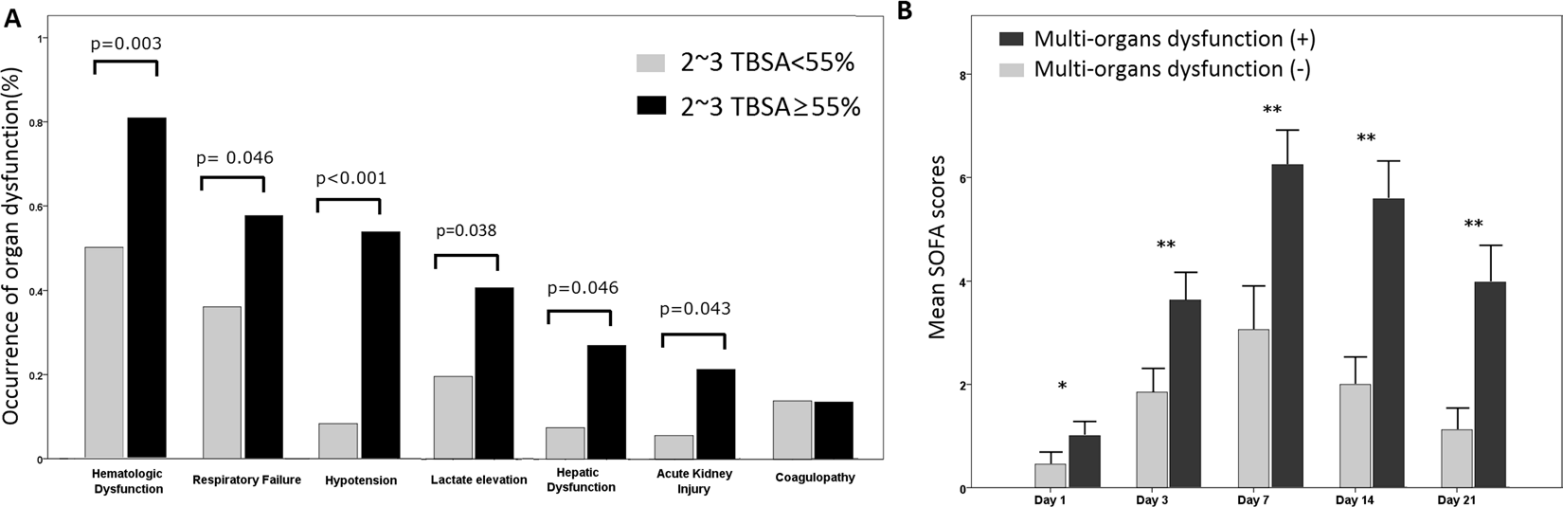
Organ dysfunction in patients with major burns on invasive ventilator support. (**A**) The occurrences of various organ dysfunctions were compared based on total body surface area (TBSA). (**B**) The sequential ogan failure asessment (SOFA) scores of patients with major burns on invasive ventilator support. The SOFA scores among patients with or without early-onset multiple organ dysfunction syndrome (MODS) on days 1, 3, 7, 14, and 21 after intubation were recorded and compared.

The proportion of patients with organ failure is shown in Table 1. The most common organ failure was hematologic failure (59/86; 68.6%), followed by respiratory failure (43/88; 48.9%) and cardiovascular failure (31/88; 35.2%). The incidence of various organ failures among patients with higher and lower TBSA is shown in Fig. 2A. Patients with a higher TBSA were more likely to present with all types of organ failure, except for coagulopathy, than those with a lower TBSA. Among these patients, 45 (51.1%) had organ dysfunction involving two or more organs within 5 days after burn injury, and they were considered to have early MODS. Patients with early MODS had a higher TBSA (67.2 ± 13.7% vs. 54.2 ± 15.2%; p < 0.001), a greater incidence of facial burn (82.2% vs. 62.8%; p = 0.041), lower serum albumin levels upon admission (1.71 ± 0.44 g/dL vs. 2.19 ± 0.82 g/dL; p = 0.002), higher sequential organ failure assessment (SOFA) scores upon admission (1.09 ± 1.26 vs. 0.47 ± 0.77; p = 0.006), and were more likely to have gram-negative bacilli detected via sputum culture (48.9% vs. 27.9%; p = 0.043) than those without early MODS. The mean number of organ failure in patients with early MODS was 3.33 ± 1.09, and that in patients without early MODS was 1.12 ± 1.10. The dynamic change in the SOFA score is shown in Fig. 2B. Patients with and without early MODS both had the highest SOFA scores on day 7 after burn injury.

### Clinical factors associated with MODS

**Figure 3:**
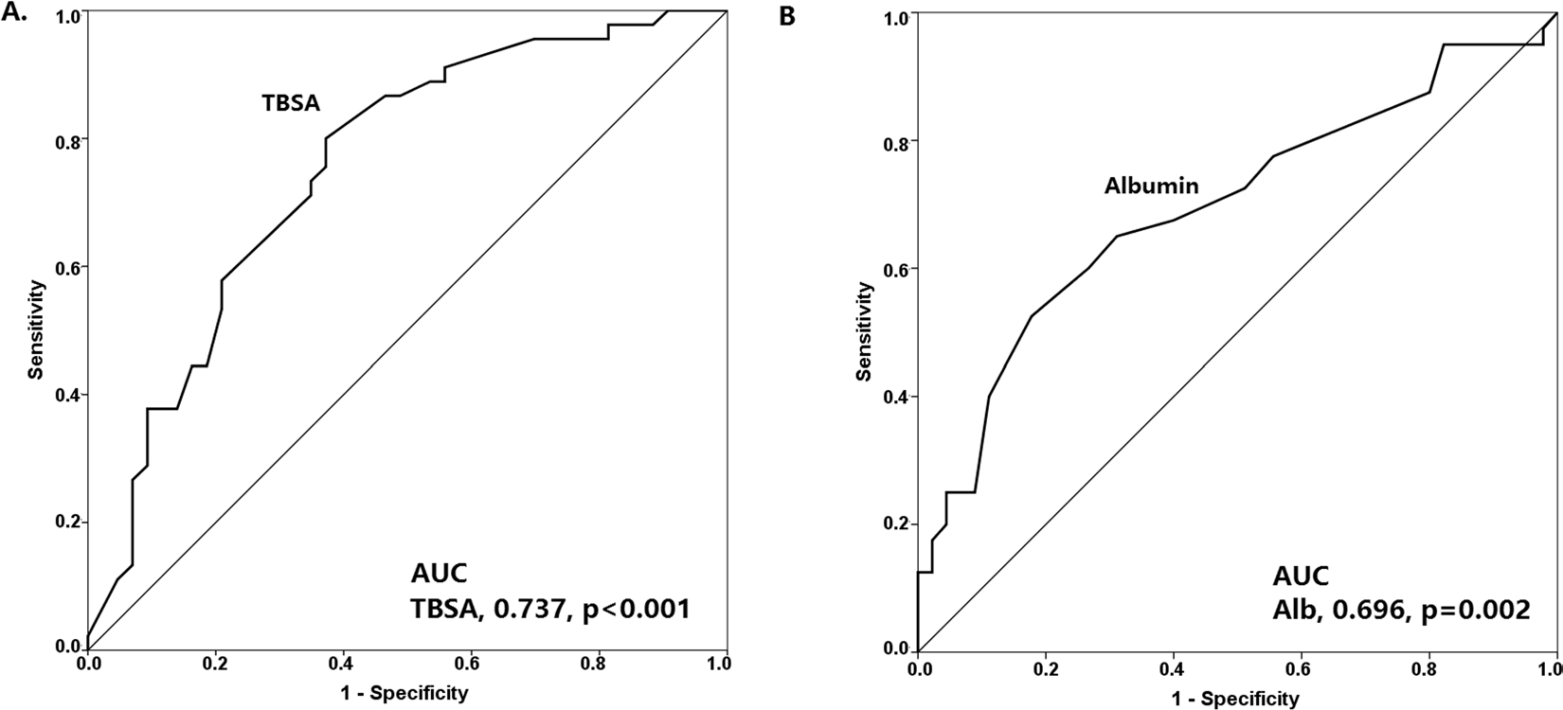
ROC curves for the prediction of early-onset multiple organ dysfunction syndrome. (**A**) The ROC curve was plotted based on total body surface area (TBSA). (**B**) The ROC curve was plotted based on serum albumin levels upon admission. AUC, area under the curve; ROC, receiver operating characteristic.

To assess the potential role of TBSA and serum albumin levels in predicting the development of early MODS, ROC curves were plotted (Fig. 3). The AUC of TBSA and serum albumin levels for early MODS were 0.737 (p < 0.001) and 0.696 (p = 0.002), respectively. The optimal calculated cut-off value for TBSA and serum albumin levels for the development of early MODS were 55% and 2.1 g/dL, respectively. Univariate and multivariate analyses were performed to identify the clinical factors associated with early MODS (Table 2). In the univariate analysis, TBSA ≥55%, the presence of facial burns, serum albumin level <2.1 g/dL upon admission, and a SOFA score ≥1 on day 1 were considered as the clinical factors associated with early MODS. In the multivariate analysis, a TBSA ≥55% (OR: 3.83; 95% CI: 1.29–11.37) and serum albumin level <2.1 g/dL upon admission (OR: 3.43; 95% CI: 1.01–11.57) were significant clinical factors associated with early MODS. Propensity-score matching analysis was also carried out to further validate the impact of TBSA and serum albumin level in patients with early MODS. As shown in Supplementary Table 1, patients with major burns who have a higher TBSA had a significantly higher risk for early MODS based on propensity-score matching for the SOFA score on day 1 and facial burn. As shown in Supplementary Table 2, patients with major burns who have a lower serum albumin level had a significantly higher risk for early MODS based on propensity-score matching for TBSA and SOFA score on day 1.

**Table 2:** Univariate and multivariate Cox-regression analysis of clinical factors associated with early MODS in patients with major burns on invasive ventilator support.

	Univaraite analysis		Multivariate analysis	
	OR (95% CI)	P value	OR (95% CI)	P value
2~3TBSA% ≥55%	6.75 (2.59–17.58)	<0.001	3.83 (1.29–11.37)	0.016
Facial burn	2.74 (1.03–7.33)	0.044	0.93 (0.24–3.53)	0.909
Serum albumin <2.1 g/dL	5.11 (1.91–13.68)	0.001	3.43 (1.01–11.57)	0.047
Day 1 SOFA ≥1	2.80 (1.18–6.65)	0.020	2.51 (0.92–6.86)	0.072

Odds ratio (OR) and 95% confidence interval (CI) were derived from the Cox proportional hazards regression model.

MODS, multiple organ dysfunction syndrome; TBSA, total body surface area; SOFA, Sequential Organ Failure Assessment.

We further analysed the proportions of early MODS in patients with various TBSA and serum albumin levels. As shown in Table 3, the proportion of early MODS in patients with TBSA ≥55% and serum albumin levels <2.1 g/dL was 76.2% (32/42). On the contrary, the proportion of early MODS in patients with TBSA <55% and serum albumin levels ≥2.1 g/dL was 18.2% (4/22). The Kaplan–Meier curves of MODS obtained after patients were stratified according to their TBSA and serum albumin level are shown in Supplemental Figure 2. Patients with higher TBSA and lower serum albumin level had the highest rate of MODS, followed by those with high TBSA and higher serum albumin level, whereas those with lower TBSA and higher albumin level had the lowest rate of MODS.

**Table 3:** The proportions of early MODS in patients with major burns on invasive ventilator support in terms of various TBSA and serum albumin levels.

	Case numbers	Early MODS		Early MODS (%)
		No, n = 43	Yes, n = 45	
TBSA% ≥ 55%, alb < 2.1 g/dL	42	10	32	76.2%
TBSA% ≥ 55%, alb ≥ 2.1 g/dL	10	6	4	40%
TBSA% < 55%, alb < 2.1 g/dL	14	9	5	35.7%
TBSA% < 55%, alb ≥ 2.1 g/dL	22	18	4	18.2%

MODS, multiple organ dysfunction syndrome; TBSA, total body surface area; alb, albumin.

### Treatment outcomes

**Figure 4:**
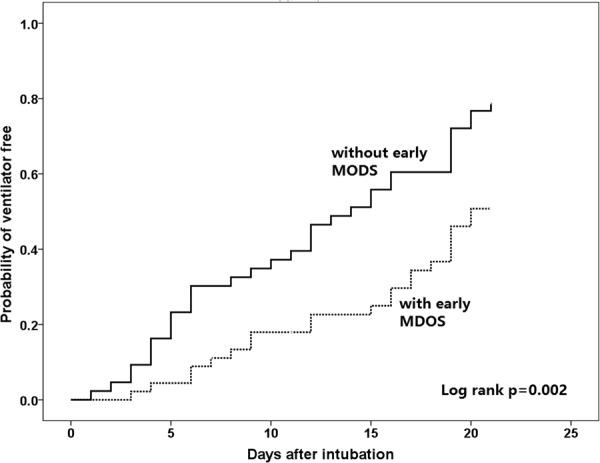
Kaplan–Meier curves of the ventilator-free status in patients with major burns on invasive ventilator support. Patients were stratified by the presence or absence of early-onset multiple organs dysfunction syndrome (MODS).

The treatment outcomes of patients with and without early MODS are shown in Table 4. The overall ventilator-free rate on days 7, 14, 21, and 28 were 19.3%, 35.2%, 63.6%, and 77.3%, respectively. Patients with early MODS had significantly lower ventilator-free rates on days 7 (p = 0.046), day 14 (p = 0.009), and day 21 (p = 0.006) than those without early MODS. These patients also had a longer length of ICU stay (56.7 ± 36.3 days vs. 42.7 ± 28 days; p = 0.045). However, the length of hospital stay was similar between the two groups. The Kaplan–Meier curves of the ventilator-free rate among patients with and without early MODS are shown in Fig. 4. Patients with early MODS had lower ventilator-free rates than those without early MODS (log-rank test, p = 0.002), and the curves separated early after intubation. The overall all-cause mortality rate was 2.3% on day 28, whereas the in-hospital mortality rate was 4.5%. No difference was observed between the groups in terms of mortality rates (Table 4).

**Table 4:** Comparisons of treatment outcomes among ventilator-supported major burn patients with and without early MODS^a^.

	Overall, n = 88	Early MODS		P value
		No, n = 43	Yes, n = 45	
Ventilator free				
At day 7	17 (19.3%)	12 (27.9%)	5 (11.1%)	0.046
At day 14	31 (35.2%)	21 (48.8%)	10 (22.2%)	0.009
At day 21	56 (63.6%)	34 (79.1%)	22 (48.9%)	0.006
At day 28	68 (77.3%)	37 (86.0%)	31 (68.9%)	0.055
Length of ICU stay	49.9 (33.0)	42.7 (28.0)	56.7 (36.3)	0.045
Length of hospital stay	93.9 (47.8)	90.9 (37.5)	105.4(49.0)	0.131
Mortality				
At day 28	2 (2.3%)	0	2 (4.4%)	0.495
In-hospital mortality	4 (4.5%)	1 (2.3%)	3 (6.7%)	0.617

^a^The data are presented as n(%) unless otherwise stated.

MODS, multiple organs dysfunction syndrome; ICU, intensive care unit.

## Discussion

To the best of our knowledge, this is the largest-scale study that enrolled 88 patients with major burns on invasive ventilator support from a single explosion event. In our study, >50% of the patients with major burns on invasive ventilator support had early MODS that occurred within 5 days after burn injury. The most common organ failure was hematologic failure (thrombocytopenia), followed by respiratory failure (poor oxygenation). In the multivariate analysis, a TBSA ≥55% and serum albumin level <2.1 g/dL upon admission were considered as independent clinical factors associated with the development of early MODS. With regard to treatment outcomes, patients with early MODS had lower ventilator-free rates and longer ICU stay. Length of hospital stay and all-cause mortality rates were similar among patients with and without early MODS.

The occurrence of organ failure is common in patients with major burns. Previous studies have reported that 27–45% of patients with burns had MODS depending on burn severity^12,13^. However, the occurrence of early MODS soon after burn injury had rarely been evaluated. The development of organ failure in patients with major burns could be multifactorial. The microvascular hyperpermeability related to burn injury can lead to tissue hypoperfusion and organ failure^16^. Large thermal injuries can induce immune suppression and cause a severe superimposed infection, followed by sepsis and organ dysfunction^15^. In this study, 62.5% of patients with major burns presented with MODS during the follow-up period, and 51.1% had early MODS that occurred within 5 days after burn injury, which is significantly higher than those in previous studies. We believe that a higher MODS rate results from severe burn. Only burn patients who required invasive ventilator support were included in our study. In contrast to the average TBSA of 44% in 499 victims from the explosion, the mean TBSA was 60.9% in our patients, and 72.7% had facial burns. Increased burn severity definitely led to the high incidence rate of MODS in this study.

The most frequently reported organ dysfunctions in patients with burns include respiratory failure, hypotension, and thrombocytopenia^12,13,21,22^. In the present study, thrombocytopenia was the most common type of organ dysfunction, followed by respiratory failure and hypotension. The incidence of inhalation injury was low in our patients, which may lead to fewer cases of severe hypoxemia. Our patients were extremely young (mean age: 21.3 years) without underlying comorbidities. Therefore, the incidence of hypotension is probably lower. The development of hematologic dysfunction ranged from 21.4–87.5% in previous studies^12,13,21,22^. A decreased platelet count within the first few days after burn injury is thought to be associated with platelet consumption secondary to burn injury or hemodilution. Late-onset thrombocytopenia is usually due to infection or sepsis^18,23^. In our study, most thrombocytopenia (71.2%, 42/59) developed within 5 days after burn injury. Therefore, platelet consumption secondary to burn injury or hemodilution was probably the major cause of thrombocytopenia in our patients.

Previous reports have shown that burn size, age, and inhalation injury are the major factors associated with the development of MODS^6,21,24^. In addition to burn size, the independent factor associated with the development of early MODS in our study included a low albumin level upon admission, which was rarely evaluated in previous studies. Hypoalbuminemia is common in patients with burns upon admission and can worsen after admission^25^. A decreased albumin level in patients with burns is multifactorial. A higher vascular permeability after burn injury can cause protein loss from the burn wounds^26^. Protein synthesis in the liver is decreased due to acute-phase response from burn injury^27^. Serum albumin level has been proposed as a risk factor for mortality in patients with burns^28^. Hypoalbuminemia upon admission is also associated with organ dysfunction in patients with burns^29^. To the best of our knowledge, this is the first study that demonstrated the significant correlation between serum albumin level and the development of early MODS. Although a recent meta-analysis suggested that albumin supplementation can improve survival during burn shock resuscitation^30^, its clinical benefit in patients with burns remains controversial^31^. However, our findings provided a clear evidence that albumin level upon admission is a convenient marker for evaluating the severity of burn injury and identifying patients who require aggressive management to prevent the development of MODS.

Results of the univariate analysis showed a significant association between higher SOFA scores on day 1 and early MODS. However, in the multivariate analysis, the independent predictors of early MODS were higher TBSA and lower serum albumin level but not SOFA scores on day 1. In our speculation, SOFA scores may represent impairment of organ function, but not necessarily MODS. On the other hand, hypoalbuminemia may result from capillary leak related to burn injury or systemic inflammatory responses, which are important in the pathogenesis of MODS. Therefore, serum albumin level is independently correlated with early MODS.

MODS is a major cause of death in patients with major burns, and the mortality rates ranged from 29% to 86% in patients who develop MODS^12,21,22^. However, the mortality rate in our patients was surprisingly low. The overall in-hospital mortality rate was 6.7% in patients with early MODS and 2.3% in those without early MODS, although their mean TBSA was 67.2% and 54.2%, respectively. The surprisingly low mortality rate was partially attributed to the low proportion of inhalation injury and the young age of our patients. Additionally, a multidisciplinary team, which consists of plastic surgeons, cardiologists, pulmonologists, intensivists, infectious disease specialists, and psychiatrists, was organized to care for the victims of the mass casualty explosion in all the participating hospitals. Finally, all the medical costs related to the burn injury was fully covered by our national health insurance. Therefore, we believe all our patients received the most aggressive management. Our results demonstrated that a significantly low mortality rate is obtainable in ventilator-supported patients with burns who developed MODS.

Despite the similarly low mortality rate among patients with and without early MODS, we found that patients with early MODS had lower extubation rates and longer ICU stay than those without early MODS. To the best our knowledge, this is the first study that focused on patients with burns on invasive ventilator support, and these patients have the most severe burns. We investigated the impact of early MODS on ventilator weaning, which has rarely been reported before. These factors made the present study exceptional. Considering the adverse impact of prolonged ventilator dependence, our findings emphasized the importance of the early recognition of patients with burns at high risk of developing MODS. Aggressive management, such as hemodynamic status optimization, infection control, and the organization of a multidisciplinary team, will be helpful to improve their treatment outcomes.

Our study had several limitations. First, this is a retrospective observational study, and some relevant clinical data related to surgical interventions for these patients were not collected and analysed in our study design. Meanwhile, we did not collect the culture results of the patients, except for those of respiratory and blood specimens. Therefore, the diagnosis of sepsis is difficult and unreliable in our patients. On the other hand, MODS would be a more objective and practical endpoint to pursue in our study design. Second, the enrolled patients were highly homogeneous because all of them were victims from a single explosion, and were young without comorbidities. Thus, our findings may not be applicable to general patients from different types of burn injury. However, it also provided us a great opportunity to observe the impact of burn on the development of early MODS without common confounding factors. Third, the relatively small sample size may predispose our analyses to type I error. However, the estimated power was 0.97 for our sample size. As a practical retrospective clinical study, we aimed at identifying the clinical predictors for early MODS. Therefore, biomarkers or novel interventions for patients with burns were not included in our study design. Finally, only ventilator-supported and ICU-admitted patients were enrolled in this study. Therefore, further studies are needed to validate our findings in patients with milder burns.

In conclusion, >50% of the patients with major burns on invasive ventilator support had early MODS that developed within 5 days after burn injury. We found that the independent clinical factors associated with early MODS were TBSA ≥55% and serum albumin level <2.1 g/dL, both of which can be used to identify high-risk patients upon admission. With regard to treatment outcomes, patients with major burns who were on invasive ventilator support and developed early MODS had a longer ICU stay and lower extubation rate than those without early MODS. Clinicians should be alert for the development of MODS in patients with major burns, particularly those with risk factors upon admission. The benefit of identification of hypoalbuminemia upon admission to manage MODS early deserves further investigation in a diverse population of major burn patients.

## Methods

### Patients and settings

This was a retrospective observational study conducted in five referral medical centres in northern Taiwan, including National Taiwan University Hospital, Tri-Service General Hospital, Chang Gung Memorial Hospital, Far Eastern Memorial Hospital, and Taipei Veterans General Hospital. The study was approved by the institutional review board of the five participating hospitals, and the informed consent was waived. All methods were performed in accordance with the relevant guidelines and regulations.

On June 27, 2015, a colour dust explosion occurred at a waterpark in northern Taiwan. A total of 499 victims suffered from this disaster, and most had major burns with a large TBSA. Their average TBSA was 44%, and 277 patients had a TBSA >40%^20^. The victims with related burn injury from the colour dust explosion and those who were admitted in the intensive care unit (ICU) or burn unit and intubated with invasive ventilator support were eligible for enrolment. Their demographic characteristics and details of burn injury, including 2nd to 3rd degree TBSA and the presence of a facial burn, were obtained from their medical charts. Fiberoptic bronchoscopy was performed if clinically indicated. The severity of inhalation injury was graded as previously reported^32^. Important clinical parameters, such as clinical presentations on the first day of burn injury, laboratory data, the use of inotropic agents, and urine output within the first 21 days of ventilator support, were also collected.

### Evaluation of organ dysfunction

Disease severities were evaluated based on the presence of organ dysfunction and SOFA scores^33^. The identification of organ dysfunction was generally based on the Surviving Sepsis Campaign criteria^34^. Briefly, respiratory dysfunction was defined as a PaO2/FiO2 ratio <200. Shock status was defined as hypotension (systolic blood pressure <90 mmHg or mean arterial pressure <70 mmHg) that required the use of vasopressors. Renal dysfunction was defined as serum creatinine level >1.5–1.9 times that at the baseline or an increase ≥0.3 mg/dL or a urine output <0.5 mL/kg/h for more than 6–12 h. Hepatic dysfunction was defined as serum bilirubin level >2 mg/dL. Hematologic dysfunction was defined as a blood platelet count <100,000/μL. Coagulopathy was defined as an international normalized ratio >1.5. Metabolic dysfunction was defined as a serum lactate level >2 mmol/L. The occurrence of organ dysfunction during the first 21 days of ventilator support was recorded and analysed. Patients with two or more organ dysfunctions were categorized as those with MODS, and MODS that occurred within 5 days after burn injury was defined as early MODS. The SOFA scores were calculated on days 1, 3, 7, 14, and 21. The dynamic changes in SOFA scores were also analysed.

### Evaluation of treatment outcomes

All patients were admitted in the ICU or burn unit after the initiation of ventilator support. A plastic surgeon managed the burn wound care, and a pulmonologist managed the ventilator support. The medical expenses related to burn wound management and ICU admission were covered in full by the national health insurance. The treatment outcomes measured in the present study included ventilator-free rates on days 7, 14, 21, and 28, length of ICU stay, length of hospital stay, mortality rate on day 28, and in-hospital mortality rate.

### Statistical analysis

The statistical analysis was performed using SPSS 20.0 software package (SPSS, Inc., Chicago, IL, USA). Categorical variables were compared using Pearson’s chi-squared test or Fisher’s exact test. Continuous variables were compared using a two-tailed independent t-test or the Mann–Whitney U test. To investigate the independent predictors of multi-organ dysfunction in patients with major burns, a multivariate Cox proportional hazards regression model with forward stepwise selection was used. A variable with p value <0.1 in the univariate analysis was used in the multivariate analysis. A receiver operating characteristic (ROC) curve was constructed to determine the predictive value of TBSA and serum albumin levels for the occurrence of early MODS, and the areas under the curve (AUC) were calculated. The optimal cut-off values of TBSA and serum albumins levels were determined accordingly. To analyse the impact of early MODS in ventilator dependence, the Kaplan–Meier method and the log-rank test were used to estimate the ventilator-free times among burn patients with and without early MODS. A p value <0.05 was considered statistically significant.

## Electronic supplementary material

Supplementary information

## References

[1] Herndon DN, Tompkins RG (2004). Support of the metabolic response to burn injury. Lancet.

[2] O’Keefe GE, Hunt JL, Purdue GF (2001). An evaluation of risk factors for mortality after burn trauma and the identification of gender-dependent differences in outcomes. J Am Coll Surg.

[3] Smith DL (1994). Effect of inhalation injury, burn size, and age on mortality: a study of 1447 consecutive burn patients. J Trauma.

[4] Colohan SM (2010). Predicting prognosis in thermal burns with associated inhalational injury: a systematic review of prognostic factors in adult burn victims. J Burn Care Res.

[5] Li H (2017). Epidemiology and outcome analysis of 6325 burn patients: a five-year retrospective study in a major burn center in Southwest China. Sci Rep.

[6] Lorente JA (2009). Organ dysfunction as estimated by the sequential organ failure assessment score is related to outcome in critically ill burn patients. Shock.

[7] Blanco J (2008). Incidence, organ dysfunction and mortality in severe sepsis: a Spanish multicentre study. Crit Care.

[8] Vincent JL, Nelson DR, Williams MD (2011). Is worsening multiple organ failure the cause of death in patients with severe sepsis?. Crit Care Med.

[9] Frohlich, M. *et al*. Epidemiology and risk factors of multiple-organ failure after multiple trauma: an analysis of 31,154 patients from the TraumaRegister DGU. *J Trauma Acute Care Surg* **76**, 921–927, discussion 927-928, 10.1097/TA.0000000000000199 (2014). 10.1097/TA.0000000000000199 24662853

[10] Manson J (2016). Early changes within the lymphocyte population are associated with the development of multiple organ dysfunction syndrome in trauma patients. Crit Care.

[11] Li N (2015). Prognostic value of natriuretic peptides in severe trauma patients with multiple organ dysfunction syndrome. Exp Ther Med.

[12] Nguyen LN, Nguyen TG (2009). Characteristics and outcomes of multiple organ dysfunction syndrome among severe-burn patients. Burns.

[13] Fitzwater J, Purdue GF, Hunt JL, O’Keefe GE (2003). The risk factors and time course of sepsis and organ dysfunction after burn trauma. J Trauma.

[14] Stanojcic M, Chen P, Xiu F, Jeschke MG (2016). Impaired Immune Response in Elderly Burn Patients: New Insights Into the Immune-senescence Phenotype. Ann Surg.

[15] Accardo-Palumbo A (2010). Reduction of plasma granzyme A correlates with severity of sepsis in burn patients. Burns.

[16] Goto M, Samonte V, Ravindranath T, Sayeed MM, Gamelli RL (2006). Burn injury exacerbates hemodynamic and metabolic responses in rats with polymicrobial sepsis. J Burn Care Res.

[17] Gomez M, Wong DT, Stewart TE, Redelmeier DA, Fish JS (2008). The FLAMES score accurately predicts mortality risk in burn patients. J Trauma.

[18] Greenhalgh DG (2007). American Burn Association consensus conference to define sepsis and infection in burns. J Burn Care Res.

[19] Bedri H (2017). A National Study of the Effect of Race, Socioeconomic Status, and Gender on Burn Outcomes. J Burn Care Res.

[20] Yang CC, Shih CL (2016). A Coordinated Emergency Response: A Color Dust Explosion at a 2015 Concert in Taiwan. Am J Public Health.

[21] Cumming J, Purdue GF, Hunt JL, O’Keefe GE (2001). Objective estimates of the incidence and consequences of multiple organ dysfunction and sepsis after burn trauma. J Trauma.

[22] Chen J (2013). Characteristics of burn deaths from 2003 to 2009 in a burn center: A retrospective study. Burns Trauma.

[23] Warner P (2011). Thrombocytopenia in the pediatric burn patient. J Burn Care Res.

[24] Ryan CM (1998). Objective estimates of the probability of death from burn injuries. N Engl J Med.

[25] Herrmann FR, Safran C, Levkoff SE, Minaker KL (1992). Serum albumin level on admission as a predictor of death, length of stay, and readmission. Arch Intern Med.

[26] Lehnhardt M (2005). A qualitative and quantitative analysis of protein loss in human burn wounds. Burns.

[27] Dickson PW, Bannister D, Schreiber G (1987). Minor burns lead to major changes in synthesis rates of plasma proteins in the liver. J Trauma.

[28] Aguayo-Becerra OA (2013). Serum albumin level as a risk factor for mortality in burn patients. Clinics (Sao Paulo).

[29] Eljaiek R, Dubois MJ (2013). Hypoalbuminemia in the first 24h of admission is associated with organ dysfunction in burned patients. Burns.

[30] Navickis RJ, Greenhalgh DG, Wilkes MM (2016). Albumin in Burn Shock Resuscitation: A Meta-Analysis of Controlled Clinical Studies. J Burn Care Res.

[31] Melinyshyn A, Callum J, Jeschke MC, Cartotto R (2013). Albumin supplementation for hypoalbuminemia following burns: unnecessary and costly!. J Burn Care Res.

[32] Endorf FW, Gamelli RL (2007). Inhalation injury, pulmonary perturbations, and fluid resuscitation. J Burn Care Res.

[33] Vincent JL (1996). The SOFA (Sepsis-related Organ Failure Assessment) score to describe organ dysfunction/failure. On behalf of the Working Group on Sepsis-Related Problems of the European Society of Intensive Care Medicine. Intensive Care Med.

[34] Dellinger RP (2013). Surviving Sepsis Campaign: international guidelines for management of severe sepsis and septic shock, 2012. Intensive Care Med.

