# Supplementary material for: Predictors of Early Onset Multiple Organ Dysfunction in Major Burn Patients with Ventilator Support: Experience from A Mass Casualty Explosion
Source: Sci Rep. 2018 Jul 19;8:10939. doi: 10.1038/s41598-018-29158-3 (PMC6053465; doi:10.1038/s41598-018-29158-3)

Title:

Predictors of Early Onset Multiple Organ Dysfunction in Major Burn Patients with Ventilator Support: Experience from A Mass Casualty Explosion

Jia-Yih Feng[1,2,3], Jung-Yien Chien[4], Kuo-Chin Kao[5, 6], Cheng-Liang Tsai[7], Fang Ming Hung[8], Fan-Min Lin[9], Han-Chung Hu[5, 6], Kun-Lun Huang[10], Chong-Jen Yu[4], Kuang-Yao Yang[1, 11,*]

[1]Department of Chest Medicine, Taipei Veterans General Hospital, Taipei, Taiwan

[2]School of Medicine, National Yang-Ming University, Taipei, Taiwan

[3]Institute of Clinical Medicine, School of Medicine, National Yang-Ming University, Taipei, Taiwan

[4]Department of Internal Medicine , National Taiwan University Hospital, National Taiwan University College of Medicine, Taipei, Taiwan

[5]Department of Thoracic Medicine, Chang Gung Memorial Hospital, Taoyuan, Taiwan

[6]Department of Respiratory Therapy, Chang Gung University, Taoyuan, Taiwan

[7]Division of Pulmonary and Critical Care, Department of Internal Medicine, Tri-Service General Hospital, National Defense Medical Center, Taipei, Taiwan

[8]Department of Surgical Intensive Care Unit, Far Eastern Memorial Hospital, New

Taipei, Taiwan

[9]Division of Pulmonary Medicine, Department of Internal Medicine,Kaohsiung Armed Forces General Hospital, Kaohsiung, Taiwan

[10]Graduate Institute of Aerospace and Undersea Medicine, National Defense Medical Center, Taipei, Taiwan

[11]Institute of Emergency and Critical Care Medicine, School of Medicine, National Yang-Ming University, Taipei, Taiwan

*Corresponding author

Supplement Table 1. Disease severities and occurrence of early MODS in major burn patients after propensity-score matching for day 1 SOFA score and facial burn[a]

	Overall, n=46	TBSA		P value
		<55%, n=23	≥55%, n=23	
Age	21.3 (4.4)	21.8 (5.1)	20.9 (3.6)	0.488
Male gender	20 (43.5%)	6 (26.1%)	12 (60.9%)	0.017
Mean 2~3TBSA%	58.9 (12.7)	49.5 (6.5)	68.3 (10.1)	<0.001
Facial burn	38 (82.6%)	19 (82.6%)	19 (82.6%)	1.000
≥2nd Smoke inhalation (n=31)[b]	10 (28.6%)	3 (18.8%)	7 (36.8%)	0.238
Serum albumin (mg/dL)	1.90 (0.68)	2.06 (0.81)	1.75 (0.50)	0.120
Organ failure				
Respiratory	18(39.1%)	7 (30.4%)	11 (47.8%)	0.227
Cardiovascular	14 (30.4%)	3 (13.0%)	11 (47.8%)	0.010
Hepatic	6 (13.0%)	2 (8.7%)	4 (17.4%)	0.665
Hematologic	34 (73.9%)	13 (56.5%)	21 (91.3%)	0.007
Renal	5 (10.9%)	2 (8.7%)	3 (13.0%)	1.000
Metabolic	17 (37.0%)	6 (26.1%)	11 (47.8%)	0.127
Coagulation	8 (17.4%)	5 (21.7%)	3 (13.0%)	0.699
Day 1 SOFA score	0.35 (0.48)	0.35 (0.49)	0.35 (0.49)	1.000
Early MODS	24 (52.2%)	6 (26.1%)	18 (78.3%)	<0.001

[a]The data are presented as n (%) unless otherwise stated.

[b]Fiberbronchoscopy is optionally performed if clinically indicated.

TBSA, total body surface area; SOFA, Sequential Organ Failure Assessment; MODS, multiple organs dysfunction syndrome

Supplement Table 2. Disease severities and occurrence of early MODS in major burn patients after propensity-score matching for TBSA and day 1 SOFA score

	Overall, n=44	Serum albumin		P value
		≥2.1g/mL, n=22	<2.1g/mL, n=22	
Age	21.1 (4.2)	21.8 (4.8)	20.5 (3.5)	0.318
Male gender	20 (45.5%)	12 (54.5%)	8 (36.4%)	0.226
Mean 2~3TBSA%	58.1 (13.9)	55.7 (13.9)	60.5 (13.9)	0.253
Facial burn	32 (72.7%)	14 (63.6%)	18 (81.8%)	0.176
≥2nd Smoke inhalation (n=31)[b]	7 (22.6%)	6 (40.0%)	1 (6.2%)	0.037
Serum albumin (mg/dL)	2.13 (0.74)	2.64 (0.70)	1.62 (0.27)	<0.001
Organ failure				
Respiratory	21(47.7%)	11 (50.0%)	10 (45.5%)	0.763
Cardiovascular	13 (29.5%)	6 (27.3%)	7 (31.8%)	0.741
Hepatic	8 (18.2%)	2 (9.1%)	6 (27.3%)	0.240
Hematologic	31 (70.5%)	14 (63.6%)	17 (77.3%)	0.322
Renal	2 (4.5%)	0	2 (9.1%)	0.488
Metabolic	14 (31.8%)	5 (22.7%)	9 (40.9%)	0.195
Coagulation	8 (18.2%)	3 (13.6%)	5 (22.7%)	0.698
Day 1 SOFA score	0.73 (1.02)	0.73 (1.03)	0.73 (1.03)	1.000
Early MODS	23 (52.3%)	8 (36.4%)	15 (68.2%)	0.035

[a]The data are presented as n (%) unless otherwise stated.

[b]Fiberbronchoscopy is optionally performed if clinically indicated.

TBSA, total body surface area; SOFA, Sequential Organ Failure Assessment; MODS, multiple organs dysfunction syndrome

Supplement figure 1. Scatter plot of TBSA and serum albumin levels in burn patients.

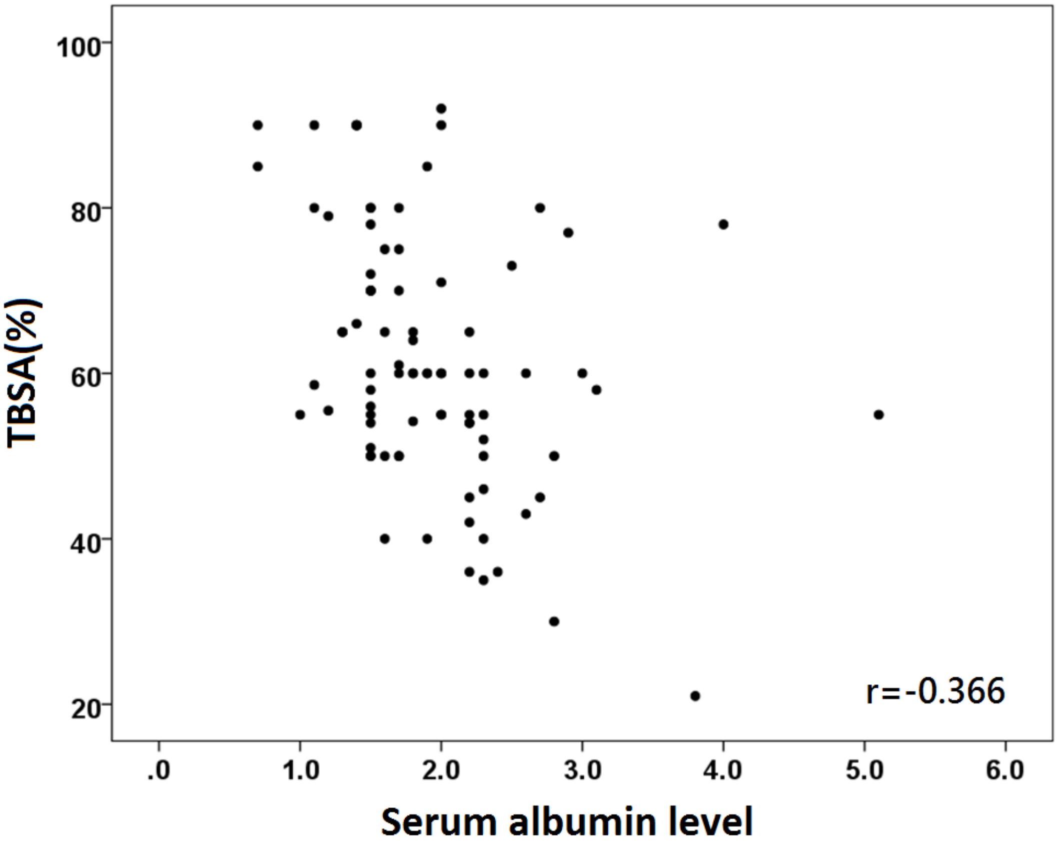

Supplement figure 2. Kaplan-Meier curves of MODS in burn patients categorize by TBSA and serum albumin level.

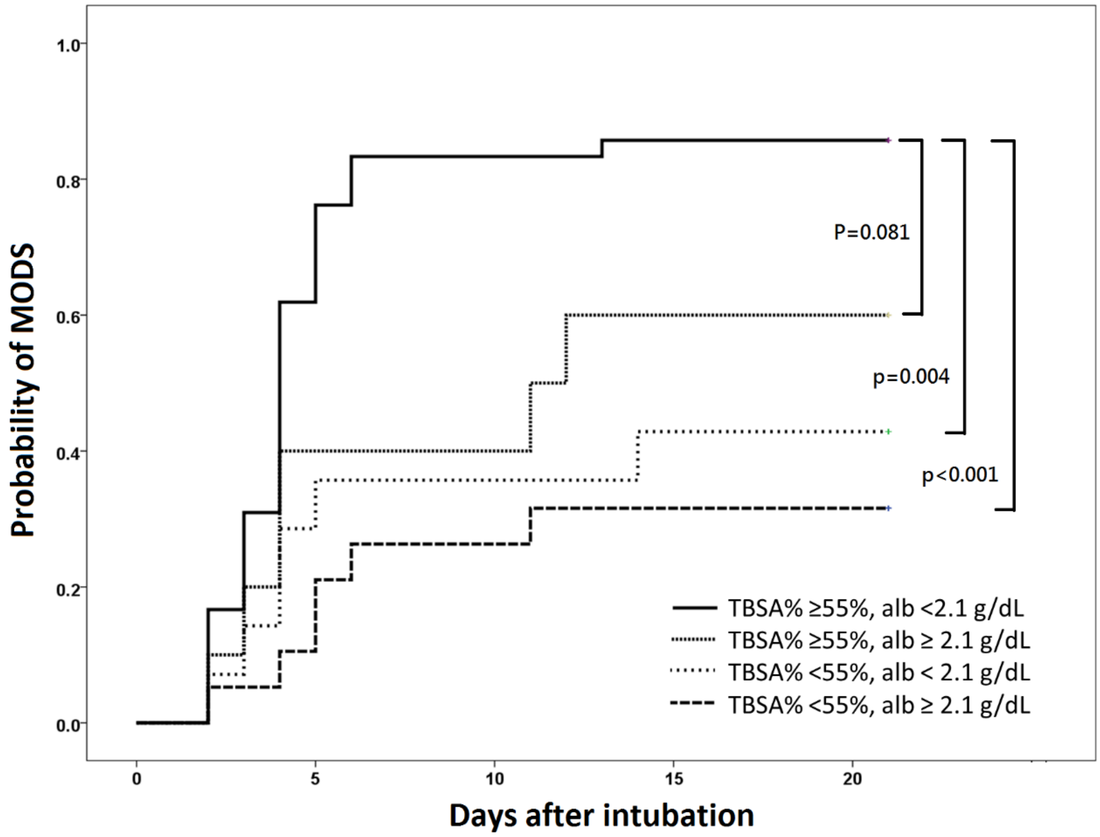

Supplement: Supplementary file 1 — Supplementary information [file 41598_2018_29158_MOESM1_ESM.pdf]
